# Factors associated with the anxiety score of deaf and hearing mothers

**DOI:** 10.1590/2317-1782/20242023239en

**Published:** 2024-08-05

**Authors:** Raquel Fabiane Nogueira, Saul Martins Paiva, Larissa Carcavalli, Ivana Meyer Prado, Mirian Castro-Braga, Lucas Guimarães Abreu, Júnia Maria Serra-Negra

**Affiliations:** 1 Departamento de Odontopediatria, Universidade Federal de Minas Gerais – UFMG - Belo Horizonte (MG), Brasil

**Keywords:** Anxiety, Behavior, Deafness, Epidemiology, Women

## Abstract

**Purpose:**

To associate maternal anxiety with sociodemographic factors, breastfeeding practices, oral habits, and the child’s entry into daycare among deaf and hearing (non-deaf) mothers.

**Methods:**

This retrospective comparative cross-sectional study included 116 mothers (29 deaf and 87 hearing) of children aged between two and five years. Deaf mothers belonged to a reference center in the city, while hearing mothers were contacted in public daycares where their children were enrolled. Mothers underwent interviews covering socio-economic factors and child development-related aspects. Additionally, they completed the Brazilian Beck Anxiety Inventory, adapted for both deaf and hearing individuals, serving as instruments to assess anxiety. The Kolmogorov-Smirnov normality test, Kruskal Wallis test, Mann-Whitney test, and Poisson Regression were employed for statistical analyses (p<0.05).

**Results:**

Deaf mothers exhibited anxiety scores one and a half times higher than hearing mothers. Moreover, mothers of children with thumb-sucking habits showed higher anxiety scores, while mothers whose children started attending daycare as infants demonstrated lower anxiety scores compared to mothers of children without such habits and who did not attend daycare.

**Conclusion:**

Deaf mothers displayed higher anxiety levels when compared to hearing mothers. Children’s behaviors, such as thumb-sucking habits, and early enrollment in daycare during the first year of life influenced maternal anxiety.

## INTRODUCTION

The motherhood desired by many women represents a major milestone of transformation and countless challenges. Physical, psychological, and professional changes can be highlighted, as these are the most impacted^(^1^)^. In the physical field, pregnancy, childbirth, and breastfeeding reflect important changes, and require understanding and learning on the part of women so that they can be experienced in a healthy way. Emotional and/or psychological changes can be caused by hormonal issues, inherent to each stage, and by the accumulation of concerns linked to the care that will be offered after childbirth^(^1^,^^2^^)^.

The various roles of women in society and the family can generate an overload, which can trigger depression and anxiety. Although anxiety is a common experience for all people, when experienced at higher levels, it can become pathological, causing harm to both physical and mental health^(^2^)^.

Maternal and child care is among the various roles played by women in society and involves fundamental actions for the full development of the child, a factor that can also be a predictor of anxiety^(^3^,^^4^^)^. The insertion of women and mothers into the job market requires this care to be shared, with the insertion of children in daycare centers being an alternative for families^(^5^)^. Only women can practice breastfeeding, and returning to work can influence the time allocated to this practice and consequently trigger specific habits in their children.

Among the most common habits among children are non-nutritive sucking (thumb sucking and/or pacifier). Finger sucking takes place inside the mother's uterus and plays the role of strengthening the perioral muscles, giving the child strength to sip breast milk during breastfeeding. This muscular work is important for perioral growth. Finger sucking also has an aspect of exploring the outside world through the mouth, which is reinforced by psychology. The pacifier is an artifact, also related to oral gratification through sucking, but its use is not recommended by the WHO, being considered an artifact that can lead to the discouragement of breastfeeding^(^6^,^^7^^)^.

Non-nutritive sucking habits, such as finger sucking and pacifiers, may originate from the baby's need for sucking that is not completely met during breastfeeding^(^7^)^. Digital sucking is part of the development of the stomatognathic system and exploration of the world by children, who tend to put objects in their mouths in their first year of life. However, culturally, there are doubts among families about the benefits and the appropriate time for finger sucking to be considered physiological^(^8^)^. Suction is relevant for the correct suction of the mother's breast during breastfeeding. The breastfeeding process, when practiced without complications, promotes intense muscular work, which results in the exhaustion of the perioral muscles and reduces the child's sucking demand^(^9^)^.

However, the practice of breastfeeding requires guidance, desire, and persistence to make it a reality^(^10^,^^11^^)^. To encourage the practice of breastfeeding, as well as maternal and child health care, it is important that women receive professional guidance and this communication needs to be effective between the parties^(^10^)^. This professional/patient communication can vary in cases of patients with disabilities.

Attention to people with disabilities has progressively become the focus of discussions and projects that enable the inclusion of all people in society. The understanding of the constitution of a society that interacts mostly through oral communication, primarily through speech, has raised important reflections regarding access to information and guidance related to health, especially for those who do not master this communicative modality. In this context, women with hearing impairment, who use visuospatial communication, raise questions regarding the preparation and capacity of health professionals to provide equitable care, which is not restricted to the capabilities inherent to communication^(^11^)^.

Hearing impairment and deafness are physiologically characterized by the lack of ability to detect, discriminate, and process environmental and speech sounds^(^12^)^. The World Health Organization (WHO) estimates that there are approximately 360 million people with disabling hearing loss. In Brazil, the latest census by the Brazilian Institute of Geography and Statistics (IBGE) identified that 5.5% of 203,080,756 Brazilians had some type of hearing impairment, and among these, 1.2% had difficulty hearing, highlighting that women had more multiple disabilities than did men^(^13^)^.

For women, the absence of hearing has a negative impact on their quality of life and daily routine, especially during pregnancy and when raising children, due to the interference in the effectiveness of interaction with people without hearing impairment^(^14^)^. One of the main factors that limits access to health information for deaf mothers is the lack of training of professionals in sign language^(^15^-^17^)^.

Given the relevance of the topic, the present study aimed to associate maternal anxiety with sociodemographic factors, breastfeeding practices, oral habits, and the child's entry into daycare, evaluating the population of deaf mothers and hearing mothers. The alternative hypothesis is that deaf mothers tend to be more anxious in caring for their children when compared to hearing mothers. The null hypothesis is that there is no difference in anxiety scores between deaf mothers and hearing mothers. The present study’s findings can encourage inclusive health promotion policies, focusing on the deaf community.

## METHODS

### Sample characteristics and study design

A retrospective cross-sectional comparative epidemiological study was developed, with a convenience sample, for which 122 mothers were contacted, of which four refused to participate and two did not meet the eligibility criteria, totaling 116 women (29 deaf and 87 hearing), mothers of children aged two to five years.

The deaf women were selected from a reference and support center for the deaf, Pastoral Care for the Deaf of Belo Horizonte (*Pastoral do Surdo de Belo Horizonte*). The female listeners were selected in the same city, in public daycare centers, located within the university campus of the Federal University of Minas Gerais (UFMG). This study used a convenience sample and was authorized by the UFMG Human Research Ethics Committee, logged under protocol no. 02371618.0.0000.5149.

### Pilot study

To test the methodology, a pilot study was developed with 20 mothers, divided into two groups (10 deaf and 10 hearing). Data collection was carried out through interviews during home visits. No changes to the methodology were necessary. Thus, participants from the pilot study were included in the main study, considering the specificity of the sample.

### Data collection

Data collection was carried out from January to December 2019, through the application of a questionnaire – the Brazilian versions for hearing and deaf individuals of the Beck Anxiety Inventory (BAI)^(^18^-^20^)^ and audiometry analysis. The research was carried out at the home of the participating mothers, after signing the Free and Informed Consent Form. Hearing mothers read and signed the written consent form. For deaf mothers, the researcher translated the consent form into Brazilian Sign Language, and then the participants signed it.

The structured questionnaire was formulated by the researchers, addressing sociodemographic issues. The questionnaire was administered through a home interview. For deaf mothers, the interview was carried out by a speech therapist (RFN) fluent in Brazilian Sign Language.

The two Brazilian versions for hearing and deaf individuals of the BAI are a self-report and a self-administered scale. It consists of 21 self-assessment items in the form of a Likert scale that varies from zero to three points (the answers to which range from “absolutely not” to “severely”). Individual scores range from 0 to 63, which allows classification into levels of anxiety intensity^(^18^,^^19^^)^.

The BAI is an instrument used to differentiate emotional and physical symptoms in people with anxiety^(^18^)^. The Brazilian versions of the BAI for deaf and hearing individuals were used. For listeners, the BAI was validated in southern Brazil^(^21^)^, by applying it at two Brazilian universities, one public and one private^(^21^)^. The Brazilian version of the BAI for the deaf was adapted by a team of researchers from São Paulo, through its application in Brazilian Sign Language for a group of deaf individuals^(^22^)^. The two versions of the BAI for deaf and hearing individuals were applied by the researchers (LC and RFN), with prior authorization from the authors, who validated the versions for Portuguese and Brazilian Sign Language. The analysis of anxiety scores received support from a psychologist at the institution.

### Eligibility criteria

Deaf and hearing mothers of children, aged two to five years, were included in the study. Deaf mothers were registered at the deaf reference center and hearing mothers were participants in the parents' meeting at their children's daycare center. Deaf mothers with severe hearing loss (above 70 and below 90 dB) and those with profound hearing loss (above 90 dB)^(^12^)^ were included. The hearing loss classification was based on the audiometry diagnosis that the mothers already had completed for registration at the reference center.

Mothers who refused to receive the researchers' home visits, mothers of children outside the age range established in the study or of syndromic children, mothers with syndromes, or those who were deaf and did not use Brazilian Sign Language were excluded.

### Variables

The mothers' anxiety score is the dependent variable in this study. Whether or not the participants are deaf is the main independent variable. Maternal sociodemographic variables (gender, age, education, marital status, profession, support network for child care, maternity leave, type of birth, and type of breastfeeding), children's non-nutritive sucking habits at the time of data collection, and children's entry into daycare are independent variables included in the analysis, which can also influence maternal anxiety.

### Statistical analysis

Statistical analysis was performed using SPSS software (Version 21.0). Descriptive statistics were performed, followed by the Kolmogorov-Smirnov normality test, to verify the distribution of anxiety scores according to the categories of the sample's independent variables. Due to the non-normal distribution of the sample, the Kruskal Wallis and Mann-Whitney tests were performed. A Poisson Regression with robust variance was also performed. Variables with a p-value ≤ 0.20 in the unadjusted model were included in the adjusted model. A significance level of 5% was considered for all statistical analyses.

### Results

The study counted on the participation of 29 deaf mothers and 87 hearing mothers. The mothers' average age was 32 years (±10.2). Our study analyzed family income quantified in minimum wage (one Brazilian minimum wage corresponds to US$242.00) and maternity leave (period of paid absence from work activities, guaranteed by Brazilian law to women after the birth of their children), the child’s development, history of non-nutritive sucking habits, and types of children's diet, based on a previous study^(^23^)^. The characterization of the sample and bivariate analysis between sociodemographic variables and children's habits are described in Table 1.

**Table 1:** Descriptive and bivariate analysis among socioeconomic, maternal, and child characteristics with mothers' level of anxiety, Brazil, 2019

**Variable**	**Frequency (%)**	**Average (SD)**	**Median [Min-Max]**	**P**
Maternal Education				
≤ 8 years	95 (81.9)	14.3 (± 11.6)	12.0 [0-50]	.179*
> 8 years	21 (18.1)	10.4 (± 09.4)	07.0 [0-27]	
Family income				
≤ US$ 484.00	76 (65.5)	14.2 (± 11.5)	12.0 [0-50]	.386*
> US$ 484.00	40 (34.5)	12.4 (± 10.8)	08.5 [0-43]	
Mother’s age				
≤ 32 years	62 (53.4)	13.9 (± 10.2)	11.0 [0-43]	.406*
≥ 33 years	54 (46.6)	13.2 (± 12.5)	09.0 [0-50]	
Deaf mothers				
Yes	29 (25.0)	18.3 (± 7.3)	18.0 [5-32]	<.001*
No	87 (75.0)	12.0 (± 12.0)	09.0 [0-50]	
Children’s order of birth				
First-born child	44 (37.9)	13.9 (± 9.3)	11.0 [0-32]	.734┼
Middle child	06 (05.2)	14.5 (± 13.5)	11.0 [1-32]	
Youngest child	35 (30.2)	12.8 (± 12.8)	09.0 [0-50]	
Only child	31 (26.7)	13.8 (± 12.1)	12.0 [0-48]	
Parents’ marital status				
Separated	49 (42.2)	12.4 (± 10.6)	11.0 [0-43]	.416*
Live together	67 (57.8)	14.4 (± 11.8)	11.0 [0-50]	
Mother’s profession				
Works at home/unemployed	35 (30.2)	15.1 (± 12.6)	12.0 [0-50]	.399*
Has a job outside the home	81 (69.8)	12.9 (± 10.7)	11.0 [0-48]	
Maternity leave				
Yes	84 (72.4)	13.1 (± 11.5)	11.0 [0-50]	.362*
No	32 (27.6)	14.7 (± 10.7)	11.5 [0-43]	
Has help to take care of the child				
Yes	73 (62.9)	13.9 (± 11.1)	12.0 [0-50]	.554*
No	43 (37.1)	12.9 (± 11.7)	09.0 [0-48]	
Exclusive care for the child				
≤ 6 months	43 (37.1)	13.6 (± 10.1)	12.0 [0-32]	.770*
≥ 7 months	73 (62.9)	13.5 (± 12.0)	10.0 [0-50]	
Type of birth				
Normal	55 (47.4)	14.8 (± 12.1)	11.0 [0-50]	.377*
^Cesarean^	61 (52.6)	12.5 (± 10.4)	09.0 [0-48]	
Mother’s age upon child birth				
≤ 19 years	15 (12.9)	16.8 (± 11.5)	14.0 [0-43]	.215*
≥ 20 years	101 (87.1)	13.1 (± 11.2)	10.0 [0-50]	
Exclusive breastfeeding				
Yes	40 (34.5)	14.7 (± 11.4)	12.5 [0-48]	.385*
No	76 (65.5)	13.0 (± 11.3)	09.5 [0-50]	
Uses a baby bottle				
Yes	81 (69.8)	14.6 (± 11.5)	12.0 [0-50]	.113*
No	35 (30.2)	11.1 (± 10.4)	09.0 [0-48]	
Uses a pacifier				
Yes	61 (52.6)	12.7 (± 11.0)	10.0 [0-50]	.358*
No	55 (47.4)	14.6 (± 12.0)	12.0 [0-48]	
Finger sucking				
Yes	07 (06.0)	23.1 (± 12.7)	27.0 [4-38]	.041*
No	109 (94.0)	12.9 (± 11.0)	10.0 [0-50]	

* Mann-Whitney test;

^┼^Kruskal Wallis test

Caption: P = probability value; SD = Standard Deviation; Min = Minimum; Max = Maximum; US$ = monthly family income converted into US dollars. Values in bold represent statistically significant associations

Table 2 shows the multivariate Poisson Regression model, considering the association between the independent variables and the maternal anxiety score. Deaf mothers showed an anxiety score of 1.551 times higher (95% CI = 1.214-1.981) when compared to hearing mothers. Mothers whose children began to attend daycare between 7 and 12 months of age showed 39% lower anxiety scores (95% CI = 0.445-0.836) when compared to mothers whose children did not attend daycare. Mothers whose children had the habit of sucking their thumbs showed an anxiety score of 1.781-fold higher (95% CI = 1.264-2.511) when compared to mothers of children who did not have the habit (Table 2).

**Table 2:** Poisson Regression Model with analysis of socioeconomic and maternal factors, children’s characteristics, and anxiety levels, Brazil, 2019

**Variables**	**Unadjusted model PR (95% CI)**	**P**	**Adjusted model PR (95% CI)**	**P**
Mother’s education				
≤ 8 years	1.366 (0.907-2.055)	.135	1.211 (0.780-1.881)	.394
> 8 years	1		1	
Family income				
≤ 2 salary in US$	1.146 (0.829-1.584)	.410		
> 2 salary in US$				
Mothers’ age (years)	0.993 (0.972-1.013)	.480		
Deaf mothers				
Yes	1.524 (1.183-1.964)	**.001**	1.551 (1.214-1.981)	**<.001**
No	1		1	
Parents’ marital status				
Separated	0.863 (0.635-1.172)	.346		
Live together	1			
Mother’s profession				
Works at home/unemployed	1.166 (0.841-1.617)	.358		
Has a job outside the home	1			
Maternity leave				
Yes	0.896 (0.656-1.223)	.490		
No	1			
Has help to take care of the child				
yes	1.078 (0.780-1.490)	.650		
No	1			
Child’s age (years)	0.985 (0.858-1.132)	.835		
Child’s sex				
Male	0.757 (0.563-1.017)	.065	0.804 (0.611-1.058)	.120
Female	1		1	
Age of the child upon entering daycare				
≤ 6 months of age	0.480 (0.279-0.827)	**.008**	0.640 (0.383-1.068)	.087
7 to 12 months of age	0.408 (0.299-0.558)	**<.001**	0.610 (0.445-0.836)	**.002**
≥ 13 months of age	0.511 (0.425-0.613)	**<.001**	0.859 (0.670-1.102)	.232
Did not go to daycare	1		1	
Uses a baby bottle				
Yes	1.322 (0.930-1.878)	.120	1.282 (0.917-1.791)	.146
No	1		1	
Uses a pacifier				
Yes	0.869 (0.643-1.174)	.361		
No	1			
Finger sucking				
Yes	1.817 (1.249-2.644)	**.002**	1.781 (1.264-2.511)	**.001**
No	1		1	

Caption: PR = prevalence ratio; CI = confidence interval; US$ = monthly family income converted into US dollars. Values in bold represent statistically significant associations; P = probability value

## DISCUSSION

When analyzing the level of maternal anxiety between deaf and hearing mothers, it was observed that deaf mothers had a higher score. To discuss the result of the higher level of anxiety among deaf mothers, it is necessary to highlight the challenges that these women face due to the fact that they have a hearing impairment.

The higher level of anxiety in deaf mothers is in line with other studies with the deaf community, which identify a positive correlation between hearing impairment and communicative limitation and high anxiety scores^(^24^,^^25^^)^. Hearing loss, in addition to depriving one of the main sensory pathways of integration with the environment, hinders the development of oral communication practiced by the majority of the population, making access to information and health care guidance restricted, creating a challenge for this population^(^14^,^^26^^)^. Women already face great pressure from society, with the role of family caregiver being delegated to them^(^2^)^. Communication challenges begin within the family nucleus^(^14^)^. When this woman becomes pregnant, the challenges faced are heightened when compared to hearing women, considering the difficulty in communication. Health professionals are trained in oral communication, so the professional support network that could provide the necessary support for this woman has weaknesses, which can generate greater anxiety^(^11^)^.

There is a lack of studies that have specifically investigated maternal and child care among deaf women; however, studies that cover such stages as the gestational or postpartum period also corroborate our findings, pointing to higher levels of anxiety^(^14^,^^26^^)^. The loss of any of the human senses is already a predisposing factor for anxiety^(^27^)^. For deaf women, the absence or restriction of the auditory sense, since childhood, constitutes numerous challenges related to insertion in a society with a predominantly oral communication, the discredit of the social and family group regarding their capabilities, and even the experience of parental overprotection, faced with risky situations throughout their lives, which become triggers in adulthood^(^25^)^.

Anxiety is a symptom that can be present in the postpartum period of hearing women, although not identified in the present study with the same score as deaf mothers. Factors such as racial-ethnic aspects, the lack of paternal support, and lower levels of education are highlighted as elements that predispose individuals to anxiety^(^28^)^. Aspects observed among deaf mothers add to the communication barriers that impact the quality of care received in both prepartum and postpartum^(^14^)^.

In the mother/child relationship, there is an exchange of emotions that can influence both mother and child. Children's habits are an example of this. In the present study, mothers of children with a thumb-sucking habit showed a higher anxiety score, once again provoking questions related to the impact of maternal emotional conditions on the child's behavior and development addressed in another study^(^29^)^.

The habit of thumb sucking is associated with issues of emotional origin and highlights the baby's lack of full satisfaction with sucking during breastfeeding, generating the need for other sources to provide the child with a sense of control and calm^(^8^)^. From another perspective, the higher anxiety score of mothers of children with such a non-nutritive sucking habit may be associated with the mother's anguish at the thought that their children may develop disharmony in their dental arches and, consequently, esthetic changes in their smile in the future^(^30^)^. It is noteworthy that this variable referred to the presence of a non-nutritive sucking habit at the time of data collection, in an age range of children between two and five years of age; therefore, the child's physiological digital sucking phase is not under analysis here^(^30^)^.

The findings of a cross-cultural study evaluating the methods used to calm the baby corroborate our results. According to the authors, there are cultures where the habit of thumb sucking is well accepted, with a cultural influence on the way society views behavior among older children^(^31^)^, an aspect that differs from Brazilian culture. Despite this, a high percentage of pacifier and/or thumb sucking habits are still observed among Brazilian children^(^32^)^. Pacifiers and baby bottles can be found in the pregnant woman's layette, reinforcing the Brazilian cultural component of these artifacts^(^32^)^. It should be noted that the habit of thumb sucking is a resource that is always accessible to the child, as the hand is a part of the body, which can make its control, and consequently its remission, difficult, which could justify the higher score of maternal anxiety^(^33^)^. If the habit continues throughout the child's life, there may be consequences for the harmonious development of the dental arches and a musculoskeletal imbalance of the stomatognathic system^(^32^)^.

The lower anxiety score among mothers of children who started daycare before the first year of life has not been addressed in other studies. However, the findings of this research highlight the importance of women entering the labor market. For women to be able to synchronize their work and family care activities, they can enroll their children in daycare centers before their first year of life. After the end of maternity leave, which in Brazil involves the child's fourth month of life, returning to work activities can cause some stress for the woman, but it can also promote a feeling of satisfaction and productivity for the woman^(^33^)^. Another favorable aspect of children entering daycare centers in the first year of life is the decentralization of concern regarding care for the children, as the mother performs other roles beyond caring for the home and family^(^33^)^. The pedagogical proposal for early childhood education, popularly known as daycare, also supports the reduction of the level of maternal anxiety, as it is recommended and guaranteed through the Law of Guidelines and Bases of National Education, of the Brazilian Ministry of Education, establishing that, from the beginning of early childhood education, the child must receive conditions that favor and stimulate their full development^(^34^)^. If the child in early childhood goes to early childhood education institutions, the mother may feel that there is a team of pedagogically reliable professionals who will take greater care of her child, a factor that may reduce her anxiety^(^35^)^.

This study is innovative in nature and brings important reflections on the promotion of maternal/child health, with inclusive actions. However, there are limitations, such as the sample size. Due to the specific nature of the participants, the number was limited and the eligibility criteria were quite specific. In addition to deafness, participants had to be female and be mothers of children between the ages of two and five, in which it was necessary to be concerned about this mother's memory bias. Mothers of older children may not remember the details of the type of breastfeeding, for example. If mothers had children younger than the selected age, there would not be a retrospective experiment. The need for previous information therefore becomes susceptible to memory bias. The proposed study design creates restrictions on deepening the identified problems, being restricted to associations, making it impossible to assess cause and effect. The scarcity of studies on this topic with deaf mothers reinforces the importance of encouraging other studies in this area, with different quantitative and qualitative designs. The results also highlight the importance of inclusive multidisciplinary work, providing necessary support for both deaf and hearing mothers. Public policies must be encouraged with an inclusive character.

## CONCLUSIONS

It can therefore be concluded that maternal anxiety was influenced by whether the mother was deaf or hearing, with a greater impact among deaf mothers. Factors related to children also influenced mothers' anxiety levels, with higher scores being observed among mothers whose children had a habit of thumb sucking and lower anxiety scores among mothers whose children entered daycare centers in the first year of life.
