# Supplementary material for: Factors associated with the anxiety score of deaf and hearing mothers
Source: Codas. 2024 Aug 5;36(5):e20230239. doi: 10.1590/2317-1782/20242023239en (PMC11340874; doi:10.1590/2317-1782/20242023239en)
Supplement: Translation

# Translation: Fatores associados ao escore de ansiedade de mães surdas e ouvintes

**DOI:** 10.1590/2317-1782/20242023239pt

**Authors:** Raquel Fabiane Nogueira, Saul Martins Paiva, Larissa Carcavalli, Ivana Meyer Prado, Mirian Castro-Braga, Lucas Guimarães Abreu, Júnia Maria Serra-Negra

**Affiliations:** 1 Departamento de Odontopediatria, Universidade Federal de Minas Gerais – UFMG - Belo Horizonte (MG), Brasil

**Keywords:** Ansiedade, Comportamento, Surdez, Epidemiologia, Mulher

## Abstract

**Objetivo:**

Associar a ansiedade materna aos fatores sociodemográficos, pratica de aleitamento, hábitos bucais e ingresso da criança em creche entre mulheres surdas e ouvintes.

**Método:**

Participaram deste estudo transversal retrospectivo comparativo, 116 mães (29 surdas e 87 ouvintes) de crianças na faixa etária entre dois e cinco anos. As mães surdas pertenciam a um centro de referência da cidade e as mães ouvintes foram contatadas em creches públicas, onde seus filhos estavam matriculados. As mães foram submetidas a entrevista sobre fatores socioeconômicos e relacionados ao desenvolvimento dos filhos, além de realizarem o preenchimento do Inventário Brasileiro de Ansiedade de Beck, nas versões para surdos e ouvintes, que foram instrumentos usados para avaliar a ansiedade. O teste de normalidade de Kolmogorov-Smirnov, os testes de Kruskal Wallis, Mann-Whitney e Regressão de Poisson foram utilizados para análises estatísticas (p &lt;0,05).

**Resultados:**

Mães surdas apresentaram escore de ansiedade uma vez e meia maior que mães ouvintes. Além disso, mães de crianças com hábito de sucção de dedo apresentaram maior escore de ansiedade e mães cujos filhos começaram a frequentar a creche ainda bebês apresentaram menor escore de ansiedade, quando comparados a crianças sem o hábito e que não frequentavam a creche.

**Conclusão:**

Mães surdas apresentaram maior ansiedade quando comparadas às ouvintes. Comportamento dos filhos com hábitos de sucção de dedo e o ingresso em creches no primeiro ano de vida influenciaram a ansiedade materna.

## INTRODUÇÃO

A maternidade almejada por muitas mulheres, representa um grande marco de transformações e inúmeros desafios. Mudanças físicas, psicológicas e profissionais podem ser destacadas, por serem as que são mais impactadas^(^1^)^. No campo físico, a gestação, parto e aleitamento materno refletem importantes mudanças e requerem entendimento e aprendizagem por parte da mulher, para que sejam vivenciadas de maneira saudável. As alterações emocionais e/ou psicológicas podem ser ocasionadas por questões hormonais, inerentes a cada etapa, e por acúmulo de preocupações ligadas aos cuidados que serão ofertados após o nascimento do filho^(^1^,^^2^^)^.

Os vários papéis da mulher na sociedade e na família, podem gerar uma sobrecarga, podendo desencadear depressão e ansiedade. Embora a ansiedade seja uma experiência comum a todas as pessoas, quando vivenciada em maiores níveis, pode atingir o caráter patológico, trazendo prejuízos à saúde física e mental^(^2^)^.

O cuidado materno infantil está entre os vários papéis desempenhados pela mulher na sociedade e envolve ações fundamentais para o pleno desenvolvimento da criança, fator que também pode ser um preditor para a ansiedade^(^3^,^^4^^)^. A inserção das mulheres e mães no mercado de trabalho exige que este cuidado seja compartilhado, sendo a inserção de crianças em creches uma alternativa para as famílias^(^5^)^. Apenas a mulher pode praticar o aleitamento materno e o retorno ao trabalho pode influenciar no tempo destinado a essa prática, e consequentemente desencadear hábitos nos filhos.

Entre os hábitos mais comuns estão os de sucção não nutritiva (sucção de dedo e/ou chupeta). A sucção digital acontece ainda dentro do útero materno e tem o papel de fortalecer a musculatura perioral dando força para a criança sorver o leite materno durante a pratica do aleitamento. Este trabalho muscular é importante para o crescimento perioral e a sucção digital também tem um aspecto de exploração do mundo exterior através da boca, reforçado pela psicologia. A chupeta é um artefato, também relacionado a gratificação oral através da sucção mas, a sua utilização não é preconizada pela OMS, sendo considerado um artefato que pode levar ao desestímulo do aleitamento materno^(^6^,^^7^^)^.

Os hábitos de sucção não nutritiva como a sucção dedo e a chupeta, podem ter origem da necessidade de sucção do bebê não suprida completamente durante o aleitamento materno^(^7^)^. A sucção digital faz parte do desenvolvimento do sistema estomatognático e exploração de mundo feito pela criança, que tende a levar objetos a boca no seu primeiro ano de vida. Entretanto, culturalmente, há dúvidas das famílias sobre os benefícios e o tempo adequado que esta sucção digital pode ser considerada fisiológica^(^8^)^. A sucção é relevante para a correta sucção no seio materno durante o aleitamento. O processo de amamentação quando praticado sem intercorrências, promove um trabalho muscular intenso, que resulta na exaustão da musculatura perioral, e diminui a demanda de sucção da criança^(^9^)^.

No entanto, a prática do aleitamento materno requer orientações, desejo e persistência, para que seja uma realidade^(^10^,^^11^^)^. Para o estímulo a prática do aleitamento materno, bem como os cuidados com a saúde materna e da criança, é importante que a mulher receba orientações profissionais e essa comunicação precisa ser eficaz entre as partes^(^10^)^. Essa comunicação profissional/paciente pode ser distinta em casos de pacientes com deficiência.

A atenção para pessoas com deficiências tem se tornado progressivamente foco de discussões e projetos que possibilitem a inclusão de todos na sociedade. O entendimento da constituição de uma sociedade que se relaciona de forma majoritária através da comunicação oral, para maior especificidade por meio da fala, tem suscitado importantes reflexões quanto ao acesso à informação e orientações relacionados à saúde, para aqueles que não dominam tal modalidade comunicativa. Neste contexto, mulheres com deficiência auditiva, que utilizam a comunicação visuoespacial, suscitam questões quanto a preparação e capacidade de profissionais da área da saúde para realização de um cuidado com equidade, que não se restringe às capacidades inerentes a comunicação^(^11^)^.

A deficiência auditiva e a surdez são caracterizadas fisiologicamente pela falta de capacidade em detectar, discriminar e processar os sons do ambiente e da fala^(^12^)^. A estimativa da Organização Mundial da Saúde é de que existem aproximadamente 360 milhões de pessoas com perda auditiva incapacitante. No Brasil, o último censo do Instituto Brasileiro de Geografia e Estatística (IBGE) identificou que 5,5% de 203.080.756 brasileiros, apresentaram algum tipo de deficiência e entre estes 1,2% apresentaram dificuldade de ouvir, destacando que mulheres tinham mais deficiências múltiplas que homens^(^13^)^.

Para as mulheres, a ausência da audição tem impacto negativo na qualidade de vida e na rotina diária, principalmente durante a gestação e criação dos filhos, devido à interferência da eficácia de interação com as pessoas sem comprometimento auditivo^(^14^)^. Um dos principais fatores que limitam esse acesso a informações em saúde para mães surdas é a falta de capacitação dos profissionais na língua de sinais^(^15^-^17^)^.

Diante da relevância do tema, o presente estudo teve como objetivo associar a ansiedade materna aos fatores sociodemográficos, prática de aleitamento, hábitos bucais e ingresso da criança em creche, avaliando a população de mães surdas e mães ouvintes. A hipótese alternativa é de que mães surdas tendem a ser mais ansiosas no cuidado dos filhos, quando comparadas as mães ouvintes. A hipótese nula é de que não existe diferença nos escores de ansiedade entre mães surdas e ouvintes. Os achados do estudo podem encorajar políticas de promoção de saúde inclusiva, com foco na comunidade surda.

## MÉTODO

### Características da amostra e desenho de estudo

Foi desenvolvido um estudo epidemiológico transversal retrospectivo comparativo, com uma amostra de conveniência para a qual foram contactadas 122 mães, dentre as quais quatro se recusaram a participar e duas não preenchiam os critérios de elegibilidade, totalizando 116 mulheres (29 surdas e 87 ouvintes), mães de crianças na faixa etária de dois a cinco anos.

As mulheres surdas foram selecionadas em um centro de referência e apoio para surdos, Pastoral do Surdo de Belo Horizonte. As mulheres ouvintes foram selecionadas na mesma cidade, em creches públicas, localizadas dentro do campus universitário da Universidade Federal de Minas Gerais. A amostra foi por conveniência. Esta Pesquisa foi autorizada pelo Comitê de Ética em Pesquisa em Humanos da Universidade Federal de Minas Gerais (protocolo nº 02371618.0.0000.5149).

### Estudo piloto

Para testar a metodologia foi desenvolvido um estudo piloto com 20 mães divididas em dois grupos (10 surdas e 10 ouvintes). A coleta de dados foi realizada por meio de entrevistas em visitas domiciliares. Não foi necessário alterações na metodologia. Desta forma, os participantes do estudo piloto foram incluídos no estudo principal, considerando a especificidade da amostra.

### Coleta de dados

A coleta de dados foi realizada de janeiro a dezembro do ano de 2019, por meio da aplicação de questionário, das versões brasileiras para ouvintes e para surdos do Inventário de Ansiedade de Beck^(^18^-^20^)^ e da análise de audiometria. A pesquisa foi realizada no domicílio das mães participantes, após assinatura do Termo de Consentimento Livre e Esclarecido. As mães ouvintes liam o termo de consentimento escrito e para as mães surdas a pesquisadora traduzia o termo de consentimento na Língua Brasileira de Sinais e depois as mesmas assinavam.

O questionário estruturado foi formulado pelos pesquisadores, abordando questões sociodemográficas. A aplicação do questionário foi por meio de entrevista domiciliar. Para as mães surdas a entrevista foi realizada por uma fonoaudióloga (RFN) fluente na Língua Brasileira de Sinais.

As duas versões brasileiras para ouvintes e para surdos do Inventário de Ansiedade de Beck (IAB), são uma escala de autorrelato e autoaplicável. É composta por 21 itens de autoavaliação na forma de escala de Likert que varia de zero a três pontos (cujas respostas vão do “absolutamente não” ao “gravemente”). Os escores individuais variam de 0 a 63, o que permite a classificação em níveis de intensidade de ansiedade^(^18^,^^19^^)^.

IAB trata-se de instrumento utilizado para diferenciar os sintomas emocionais e físicos em pessoas com ansiedade^(^18^)^. Foram utilizadas as versões brasileiras do IAB para surdos e para ouvintes. Para ouvintes, o IAB foi validado no sul do Brasil^(^21^)^, mediante aplicação do Inventário em duas universidades brasileiras, uma pública e uma privada^(^21^)^. A versão brasileira do IAB para surdos foi adaptada por uma equipe de pesquisadores paulistas, através da sua aplicação na Língua Brasileira de Sinais para um grupo de indivíduos surdos^(^22^)^. As duas versões do IAB para surdos e ouvintes foram aplicadas pelas pesquisadoras (LC e RFN), com autorização prévia dos autores que validaram as versões para o português e para Libras. A análise dos escores de ansiedade receberam suporte de uma psicóloga da instituição.

### Critérios de elegibilidade

Foram incluídas no estudo mães surdas e ouvintes de crianças na faixa etária de dois a cinco anos. As mães surdas eram cadastradas no centro de referência de surdos e mães ouvintes eram participantes da reunião de pais na creche dos filhos. Foram incluídas mães surdas com perda auditiva severa (acima de 70 e abaixo de 90 dB) e aquelas com perda auditiva profunda (acima de 90 dB)^(^12^)^. A classificação de perda auditiva baseou-se no diagnóstico por audiometria que as mães já possuíam para cadastro no centro de referência.

Foram excluídas mães que recusaram a visita domiciliar das pesquisadoras, mães de crianças fora da faixa etária estabelecida no estudo ou sindrômicas, mães com síndromes ou surdas que não utilizavam a Língua Brasileira de Sinais.

### Variáveis

O escore de ansiedade das mães é a variável dependente deste estudo. A surdez ou não das participantes é variável independente principal. As variáveis sociodemográficas maternas (gênero, idade, escolaridade, estado civil, profissão, rede de apoio para cuidado dos filhos, licença maternidade, tipo de parto e tipo de aleitamento), os hábitos de sucção não nutritiva dos filhos no momento da coleta de dados e o ingresso dos filhos em creche são variáveis independentes incluídas na análise, que também podem influenciar na ansiedade materna.

### Análise estatística

A análise estatística foi realizada utilizando o software SPSS (Versão 21.0). Foram realizadas estatísticas descritivas, seguidas do teste de normalidade de Kolmogorov-Smirnov, para verificar a distribuição dos escores de ansiedade de acordo com as categorias das variáveis independentes da amostra. Devido à distribuição não normal da amostra, foram realizados os testes de Kruskal Wallis e Mann-Whitney. Também foi realizada uma Regressão de Poisson com variância robusta. Variáveis com um valor-p ≤ 0,20 no modelo não ajustado foram incluídas no modelo ajustado. Um nível de significância de 5% foi considerado para todas as análises estatísticas.

### Resultados

Participaram do estudo 29 mães surdas e 87 mães ouvintes. A média de idade das mães foi de 32 anos (± 10,2). Foram analisadas a renda familiar quantificada em salário-mínimo (um salário-mínimo brasileiro corresponde a US$242,00) e a licença maternidade (período de afastamento remunerado das atividades de trabalho, garantido pela lei brasileira a mulheres após o nascimento dos filhos), desenvolvimento da criança, histórico de hábitos de sucção não nutritiva e tipos de alimentação dos filhos, baseado em estudo anterior^(^23^)^. A caracterização da amostra e análise bivariada entre as variáveis sociodemográficas e hábitos dos filhos estão descritas na Tabela 1.

**Tabela 1:** Análise descritiva e bivariada entre características socioeconômicas, maternas e infantis com nível de ansiedade das mães, Brasil, 2019

**Variáveis**	**Frequência (%)**	**Média (DP)**	**Mediana [Min-Max]**	**P**
Educação materna				
≤ 8 anos	95 (81,9)	14,3 (± 11,6)	12,0 [0-50]	,179*
&gt; 8 anos	21 (18,1)	10,4 (± 09,4)	07,0 [0-27]	
Renda familiar				
≤ US$ 484,00	76 (65,5)	14,2 (± 11,5)	12,0 [0-50]	,386*
&gt; US$ 484,00	40 (34,5)	12,4 (± 10,8)	08,5 [0-43]	
Idade da mãe				
≤ 32 anos	62 (53,4)	13,9 (± 10,2)	11,0 [0-43]	,406*
≥ 33 anos	54 (46,6)	13,2 (± 12,5)	09,0 [0-50]	
Mães surdas				
Sim	29 (25,0)	18,3 (± 7,3)	18,0 [5-32]	&lt;,001*
Não	87 (75,0)	12,0 (± 12,0)	09,0 [0-50]	
Ordem nascimento de filhos				
Primogênito	44 (37,9)	13,9 (± 9,3)	11,0 [0-32]	,734┼
Filho do meio	06 (05,2)	14,5 (± 13,5)	11,0 [1-32]	
Caçula	35 (30,2)	12,8 (± 12,8)	09,0 [0-50]	
Filho único	31 (26,7)	13,8 (± 12,1)	12,0 [0-48]	
Estado civil dos pais				
Vivem Separados	49 (42,2)	12,4 (± 10,6)	11,0 [0-43]	,416*
Vivem juntos	67 (57,8)	14,4 (± 11,8)	11,0 [0-50]	
Profissão da mãe				
Trabalha em casa/não tem emprego	35 (30,2)	15,1 (± 12,6)	12,0 [0-50]	,399*
Tem emprego fora de casa	81 (69,8)	12,9 (± 10,7)	11,0 [0-48]	
Licença maternidade				
Sim	84 (72,4)	13,1 (± 11,5)	11,0 [0-50]	,362*
Não	32 (27,6)	14,7 (± 10,7)	11,5 [0-43]	
Tem ajuda para cuidar do filho				
Sim	73 (62,9)	13,9 (± 11,1)	12,0 [0-50]	,554*
Não	43 (37,1)	12.9 (± 11.7)	09.0 [0-48]	
Cuidado exclusivo para a criança				
≤ 6 meses	43 (37,1)	13,6 (± 10,1)	12,0 [0-32]	,770*
≥ 7 meses	73 (62,9)	13,5 (± 12,0)	10,0 [0-50]	
Tipo de parto				
Normal	55 (47,4)	14,8 (± 12,1)	11,0 [0-50]	,377*
Cesariana	61 (52,6)	12,5 (± 10,4)	09,0 [0-48]	
Idade da mãe no nascimento do filho				
≤ 19 anos	15 (12,9)	16,8 (± 11,5)	14,0 [0-43]	,215*
≥ 20 anos	101 (87,1)	13,1 (± 11,2)	10,0 [0-50]	
Aleitamento exclusivo				
Sim	40 (34,5)	14,7 (± 11,4)	12,5 [0-48]	,385*
Não	76 (65,5)	13,0 (± 11,3)	09,5 [0-50]	
Uso de mamadeira				
Sim	81 (69,8)	14,6 (± 11,5)	12,0 [0-50]	,113*
Não	35 (30,2)	11,1 (± 10,4)	09,0 [0-48]	
Uso de chupeta				
Sim	61 (52,6)	12,7 (± 11,0)	10,0 [0-50]	,358*
Não	55 (47,4)	14,6 (± 12,0)	12,0 [0-48]	
Hábito de sucção de dedo				
Sim	07 (06,0)	23,1 (± 12,7)	27,0 [4-38]	,041*
Não	109 (94,0)	12,9 (± 11,0)	10,0 [0-50]	

* Mann-Whitney teste;

^┼^Kruskal Wallis teste

Legenda: P = valor de probabilidade; DP = Desvio Padrão; Min = Mínimo; Max = Máximo; US$ = renda mensal familiar convertida em dólar americano. Valores em negrito representam associações estatisticamente significativas

Na Tabela 2 observa-se o modelo multivariado da Regressão de Poisson considerando a associação entre as variáveis independentes e o escore de ansiedade materno. Mães surdas apresentaram escore de ansiedade 1,551 vezes maior (IC 95% = 1,214-1,981) em comparação as mães ouvintes. Mães cujos filhos começaram a frequentar a creche entre 7 e 12 meses de idade tiveram escore de ansiedade 39% menor (95% IC = 0,445-0,836) em comparação a mães cujos filhos não frequentavam a creche. Mães cujos filhos tinham o hábito de chupar o dedo tiveram escore de ansiedade 1,781 vezes maior (95% IC = 1,264-2,511) em comparação as mães de crianças que não tinham o hábito (Tabela 2).

**Tabela 2:** Modelo de Regressão de Poisson com análise dos fatores socioeconômicos, maternais, características das crianças e níveis de ansiedade, Brasil, 2019

**Variáveis**	**Modelo não ajustado RP (95% IC)**	**P**	**Modelo ajustado RP (95% IC)**	**P**
Escolaridade das mães				
≤ 8 anos	1,366 (0,907-2,055)	,135	1,211 (0,780-1,881)	,394
&gt; 8 anos	1		1	
Renda familiar				
≤ 2 salários em US$	1,146 (0,829-1,584)	,410		
&gt; 2 salários em US$				
Idade das mães (anos)	0,993 (0,972-1,013)	,480		
Mães surdas				
Sim	1,524 (1,183-1,964)	**,001**	1,551 (1,214-1,981)	**&lt;.001**
Não	1		1	
Estado civil dos pais				
Vivem separados	0,863 (0,635-1,172)	,346		
Vivem juntos	1			
Profissão da mãe				
Trabalha em casa/não tem emprego	1,166 (0,841-1,617)	,358		
Tem emprego fora de casa	1			
Licença maternidade				
Sim	0,896 (0,656-1,223)	,490		
Não	1			
Tem ajuda para cuidar do filho				
Sim	1,078 (0,780-1,490)	,650		
Não	1			
Idade da criança (anos)	0,985 (0,858-1,132)	,835		
Sexo da criança				
Masculino	0,757 (0,563-1,017)	,065	0,804 (0,611-1,058)	,120
Feminino	1		1	
Idade da criança ao ingressar em creches				
≤ 6 meses de idade	0,480 (0,279-0,827)	**,008**	0,640 (0,383-1,068)	,087
7 to 12 meses de idade	0,408 (0,299-0,558)	**&lt;,001**	0,610 (0,445-0,836)	**,002**
≥ 13 meses de idade	0,511 (0,425-0,613)	**&lt;,001**	0,859 (0,670-1,102)	,232
Não foi para creche	1		1	
Uso de mamadeira				
Sim	1,322 (0,930-1,878)	,120	1,282 (0,917-1,791)	,146
Não	1		1	
Uso de chupeta				
Sim	0,869 (0,643-1,174)	,361		
Não	1			
Hábito de sucção de dedo				
Sim	1,817 (1,249-2,644)	**,002**	1,781 (1,264-2,511)	**,001**
Não	1		1	

Legenda: RP = razão de prevalência; IC = intervalo de confiança; US$ = renda mensal familiar convertida em dólar americano. Valores em negrito representam associações estatisticamente significativas; P = valor de probabilidade

## DISCUSSÃO

Ao analisar o nível de ansiedade materno entre mães surdas e mães ouvintes, observou-se que mães surdas apresentaram maior escore. Para discutir o resultado do maior nível de ansiedade entre mães surdas faz-se necessário destacar os desafios que estas mulheres enfrentam pelo fato de apresentarem a deficiência.

O maior nível de ansiedade em mães surdas vai ao encontro de outros estudos com a comunidade surda, que identifica uma correlação positiva entre o comprometimento auditivo e a limitação comunicativa e o escore elevado de ansiedade^(^24^,^^25^^)^. A perda auditiva, além de privar uma das principais vias sensoriais de integração com o meio, dificulta o desenvolvimento da comunicação oral, praticada pela maior parte da população, tornando restrito o acesso a informações e orientações de cuidado à saúde, determinando um desafio para esta população^(^14^,^^26^^)^. A mulher já enfrenta grandes pressões da sociedade, sendo delegada a ela o papel de cuidadora da família^(^2^)^. Ainda dentro do núcleo familiar iniciam os desafios de comunicação^(^14^)^. Quando essa mulher engravida, os desafios enfrentados se potencializam quando comparadas a mulheres ouvintes, considerando a dificuldade de comunicação. Os profissionais de saúde são treinados para a comunicação oral, de modo que a rede de apoio profissional que poderia prover o suporte necessário para esta mulher, apresenta fragilidades, o que pode gerar mais ansiedade^(^11^)^.

Há uma escassez de estudos que investigaram especificamente a realização do cuidado materno infantil entre mulheres surdas, no entanto, estudos que contemplam etapas como o período gestacional ou puerpério, também corroboram nossos achados, apontando para níveis mais elevados de ansiedade^(^14^,^^26^^)^. A perda de qualquer dos sentidos humanos já é um fator predispor da ansiedade^(^27^)^. Para as mulheres surdas a ausência ou restrição do sentido auditivo, constitui desde a sua infância inúmeros desafios relacionados a inserção em uma sociedade de comunicação com predomínio oral, o descrédito do grupo social e familiar quanto a suas capacidades, e até mesmo a experiência de superproteção dos pais, frente a situações de risco ao longo da vida, tornando-se gatilhos na fase adulta^(^25^)^

A ansiedade é um sintoma que pode ser presente no puerpério de mulheres ouvintes, embora não identificada no presente estudo com o mesmo escore das mães surdas, os fatores como aspectos étnicos raciais, ausência de suporte paterno e menor nível de escolaridade são apontados como elementos que predispõe a ansiedade^(^28^)^. Aspectos observados entre as mães surdas somado as barreiras da comunicação que impactam na qualidade do atendimento recebido no pré e pós parto^(^14^)^.

Na relação mãe/filho há uma troca de emoções que pode influenciar tanto a mãe quanto a criança. Os hábitos dos filhos são um exemplo disso. No presente estudo, mães de filhos com o hábito de sucção de dedo apresentaram maior escore de ansiedade, incitando novamente os questionamentos relacionados ao impacto das condições emocionais maternas sobre o comportamento e desenvolvimento da criança abordado em outro estudo^(^29^)^. O hábito de sucção digital está associado a questões de origem emocional, e retoma a atenção à falta da satisfação plena do bebê com a sucção durante o aleitamento, gerando a necessidade de outras fontes para sensação de controle e calmaria para a criança^(^8^)^. Por outra ótica, o maior escore de ansiedade das mães de filhos com tal hábito de sucção não nutritiva, pode estar associado a angústia da mãe ao pensar que os filhos podem desenvolver desarmonias nas arcadas dentárias e consequentemente alterações estéticas do sorriso no futuro^(^30^)^. Destaca-se que esta variável referia-se à presença do hábito de sucção não nutritiva no momento da coleta de dados, numa faixa etária de crianças entre dois e cinco anos, portanto, não está em análise a fase da sucção digital fisiológica da criança^(^30^)^.

Os achados de um estudo transcultural avaliando os métodos utilizados para acalmar o bebê corroboram com nossos resultados. Segundo os autores, existem culturas onde o hábito de sugar o dedo é bem aceito, havendo uma influência cultural na forma como a sociedade encara o comportamento entre crianças mais velhas^(^31^)^, aspecto este que difere da cultura brasileira. Apesar disso, observa-se um alto percentual dos hábitos de sucção de chupeta e/ou de dedo entre crianças brasileiras^(^32^)^. As chupetas e mamadeiras podem ser encontradas no enxoval da gestante, reforçando o componente cultural brasileiro destes artefatos^(^32^)^. Há de se ressaltar que o hábito de sucção de dedo é um recurso que encontra-se sempre acessível à criança, por ser a mão uma parte do corpo, o que pode dificultar seu controle e consequentemente sua remição, o que poderia justificar o maior escore de ansiedade materno^(^33^)^.Caso o hábito prolongue-se na vida da criança, podem haver consequências para o desenvolvimento harmonioso das arcadas dentárias e um desequilíbrio musculoesquelético do sistema estomatognático^(^32^)^.

O menor escore de ansiedade entre mães de crianças que iniciaram na creche antes do primeiro ano de vida, não foi abordado em outros estudos. No entanto, os achados desta pesquisa destacam a importância da mulher na inserção no mercado laboral. Para que a mulher consiga sincronizar suas atividades de trabalho e de cuidados com a família, ela pode utilizar do recurso de matricular seu filho em creches antes do primeiro ano de vida. Após a finalização da licença maternidade, que no Brasil envolve o quarto mês de vida do filho, o retorno às atividades laborais pode causar certo estresse para a mulher, mas, também, promove um sentimento de satisfação e produtividade para ela^(^33^)^. Outro aspecto favorável do ingresso da criança em creches ainda no primeiro ano de vida, é a descentralização da preocupação nos filhos, por meio da execução de outros papéis pela mãe, para além dos cuidados com a casa e a família^(^33^)^. A proposta pedagógica para a educação infantil, popularmente conhecida como creche, também vai ao encontro a redução do nível de ansiedade materna, visto que é preconizado e garantido por meio da Lei de Diretrizes e Bases da Educação Nacional, do Ministério da Educação do Brasil, que desde o início da educação infantil, a criança deve receber condições que favoreça e estimule o seu desenvolvimento integral^(^34^)^. Se a criança na primeiríssima infância vai para instituições de educação infantil, a mãe pode sentir que há uma equipe de profissionais pedagogicamente confiáveis, que terão maior cuidado com sua criança e este pode ser um fator que diminua a sua ansiedade^(^35^)^.

Este estudo tem caráter inovador e traz reflexões importantes na promoção da saúde materno/infantil, com ações inclusivas. Entretanto, há limitações, como o tamanho da amostra. Devido a especificidade das participantes, o número foi limitado e os critérios de elegibilidade eram também muito específicos. Além da surdez, a participantes deveriam ser do sexo feminino e ser mãe de crianças na faixa etária de dois a cinco anos, onde fez-se necessária a preocupação com o viés de memória desta mãe. Mães de crianças mais velhas poderiam não ter a lembrança dos detalhes do tipo de aleitamento, por exemplo. Se as mães tivessem filhos com idade inferior a selecionada, não haveria uma experiência retrospectiva. A necessidade de informações pregressas torna-se suscetível ao viés de memória. O desenho de estudo proposto gera restrição ao aprofundamento em relação às problemáticas identificadas, sendo restrito a associações, não sendo possível avaliar causa e efeito. A escassez de estudos dentro desta temática com mães surdas, reforça a importância de estimular outros estudos nessa área, com diferentes delineamentos tanto quantitativo quanto qualitativo. Os resultados também ressaltam a importância do trabalho multidisciplinar inclusivo, dando suporte necessário para as mães, tanto surdas quanto ouvintes. Políticas públicas devem ser encorajadas com caráter inclusivo.

## CONCLUSÕES

Conclui-se que a ansiedade materna sofreu influência no fato da mãe ser surda ou ouvinte, havendo maior impacto entre as mães surdas. Fatores relacionados aos filhos também influenciaram o nível de ansiedade das mães, sendo observado maior escore entre mães cujos filhos tinham hábito de sucção digital e menores escores de ansiedade entre mães cujos filhos ingressaram em creches no primeiro ano de vida.
